# Tailor-Made Deep Eutectic Solvents for Simultaneous Extraction of Five Aromatic Acids from *Ginkgo biloba* Leaves

**DOI:** 10.3390/molecules23123214

**Published:** 2018-12-05

**Authors:** Jun Cao, Huimin Wang, Wei Zhang, Fuliang Cao, Geli Ma, Erzheng Su

**Affiliations:** 1 Department of Food Science and Technology, College of Light Industry and Food Engineering, Nanjing Forestry University, Nanjing 210037, China; JuneC75@njfu.edu.cn (J.C.); huiminwang@njfu.edu.cn (H.W.); ZWahoo@njfu.edu.cn (W.Z.); 2 Co-Innovation Center for the Sustainable Forestry in Southern China, College of Forestry, Nanjing Forestry University, Nanjing 210037, China; flcao@njfu.edu.cn; 3 School of Food and Biological Engineering, Zhengzhou University of Light Industry, Zhengzhou 450002, China; mageli@zzuli.edu.cn; 4 State Key Laboratory of Natural Medicines, China Pharmaceutical University, Nanjing 210009, China

**Keywords:** deep eutectic solvents, *Ginkgo biloba* leaves, simultaneous extraction, shikimic acids, 6-hydroxykynurenic acid

## Abstract

*Ginkgo biloba* leaves have various health benefits due to the presence of bioactive compounds such as polyprenyl acetates, flavonoids, and terpene trilactones. However, there is little literature reported on the aromatic acids in *Ginkgo biloba* leaves. In this work, five aromatic acids including shikimic acid (SA), 6-hydroxykynurenic acid (6-HKA), protocatechuic acid (PA), gallic acid (GAA), and *p*-hydroxybenzoic acid (PHBA) were simultaneously extracted from *Ginkgo biloba* leaves by employing the green deep eutectic solvents (DESs). A DES tailor-made from xylitol, glycolic acid and 1,5-pentanedioic acid at a molar ratio of 1:3:1 with 50% (*w*/*w*) water addition, named as NGG50, gave higher extraction yields for the five aromatic acids. Main factors affecting the extraction process were further optimized. The highest extraction yields of SA, GAA, 6-HKA, PA, and PHBA were 94.15 ± 0.96 mg/g, 332.69 ± 5.19 μg/g, 25.90 ± 0.61 μg/g, 429.89 ± 11.47 μg/g and 67.94 ± 0.37 μg/g, respectively. The NGG50-based extraction process developed here was a successful attempt of simultaneously extracting five aromatic acids from *Ginkgo biloba* leaves for the first time, which could provide a new exploitation direction of *Ginkgo biloba* leaves.

## 1. Introduction

**Figure 1:**
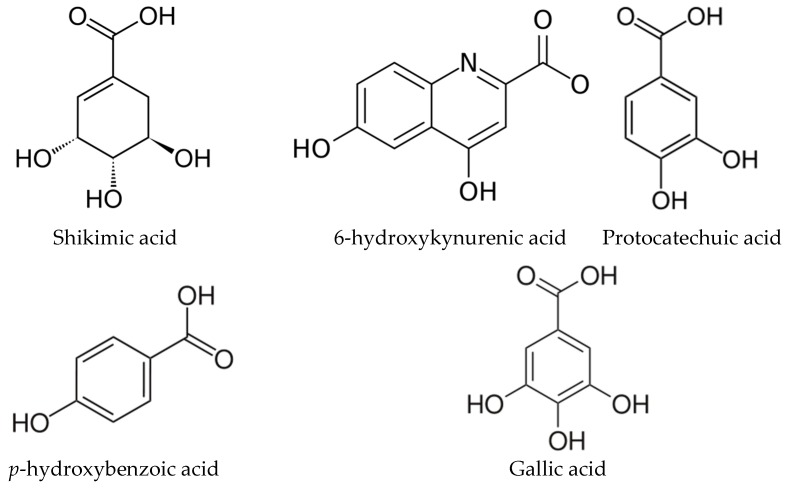
Chemical structures of five aromatic acids.

*Ginkgo biloba*, gymnosperm belongs to ginkgopsida, is mainly cultivated in China. Both its leaves and fruits have various health benefits, such as treating cerebral thrombosis and preventing cardiovascular diseases, due to the presence of bioactive compounds [1,2]. The bioactive compounds in *Ginkgo biloba* leaves have been extensively studied by using modern separation and extraction techniques, and over 170 kinds of bioactive compounds have been found. However, most of the studies focused on flavonoids, terpene trilactones and ginkgolic acid, ignoring the aromatic acids in *Ginkgo biloba* leaves [2,3,4]. The main aromatic acids in *Ginkgo biloba* leaves are shikimic acid, 6-hydroxykynurenic acid, protocatechuic acid, *p*-hydroxybenzoic acid and gallic acid (Figure 1) [5]. In addition, trace amounts of caffeic acid, *p*-coumaric acid, vanillic acid, ferulic acid, and chlorogenic acid also exist in *Ginkgo biloba* leaves.

Shikimic acid (SA) plays a key role in the biosynthesis of many important natural products, which is so important that one of the key biosynthetic pathways is referred to as the shikimate pathway [6]. It is also widely used as an important antiviral agent for the H_5_N_1_ strain of influenza [7]. The main plant source of SA is star anise, which can be harvested only in March and May. The potential alternative source of SA is urgently required due to the growing demand for pharmaceuticals. 6-hydroxykynurenic acid (6-HKA), a rare type of nitrogen-containing phenolic acid, is the only antagonist of the amino acid neurotransmitter in the central nervous system, acting on the *N*-methyl-d-aspartic acid to relieve cerebral hypoxia symptoms [8]. 6-HKA has high affinity to the α-amino-3-hydroxy-5-methyl-4-isoxazole propionate (AMPA) receptor and plays a role in the brain tissue under the impaired blood-brain barrier integrity. According to the previous report [9], *Ginkgo biloba* leaves is the only plant resource of the 6-HKA except for *Nicotiana tabacum*. Protocatechuic acid (PA) may be efficient to treat the oxidative stress-induced neurodegenerative disease due to increasing the cellular viability of pheochromocytoma cell and markedly attenuating H_2_O_2_-induced apoptotic cell death [10]. PA could also induce the apoptosis of HL60 leukemia cell and HepG2 hepatocellular carcinoma [11]. The biological activities of gallic acid (GAA) and *p*-hydroxybenzoic acid (PHBA) have also been widely studied, such as anti-oxidative, tumor-inhibition and anti-inflammation.

Although the aromatic acids with biological activities were found in *Ginkgo biloba* leaves, there is little literature focused on the extraction of the aromatic acids [7,9]. Considering that the polarities of the above-referred aromatic acids are similar, they can be detected simultaneously under the same analysis conditions. Therefore, we wondered if there is a solvent that could efficiently extract these aromatic acids simultaneously.

Deep eutectic solvents (DESs) are a kind of green solvents that could change their physical and chemical properties through adjusting the composition [12]. DESs can be prepared in high purity expediently by mixing starting materials at a certain molar ratio, which are acted as hydrogen bond donors (HBDs) and hydrogen bond acceptors (HBAs). DESs have been extensively studied for the extraction of natural bioactive compounds, with the advantages of low cost, high efficiency and good environmental compatibility [13,14,15]. In our laboratory, tailor-made DESs had been employed as extraction solvents to extract polyprenyl acetates and terpene trilactones from *Ginkgo biloba* leaves successfully [16,17]. Therefore, DESs may also be tailor-made to extract aromatic acids from *Ginkgo biloba* leaves simultaneously.

In this work, we attempted to provide a cleaner and simultaneous extraction for multiple aromatic acids from *Ginkgo biloba* leaves with DESs as green solvents, which could greatly reduce the energy consumption and wastes production. For this purpose, SA, PA, 6-HKA, GAA and PHBA were selected as the target aromatic acids because their detectable contents in *Ginkgo biloba* leaves. After the method for analysis of the five aromatic acids was established, the tailoring of DESs for higher extraction efficiency was demonstrated. Following that, the extraction methods and conditions relevant to the extraction efficiency were optimized systematically.

## 2. Materials and Methods

### 2.1. Materials

The *Ginkgo biloba* leaves picked on May were purchased from Chinese Herb Transaction Center (Bozhou, China). The leaves were dried at 40 °C to constant weight in vacuum oven, and then pulverized to 30–40 mesh by a disintegrator. The water content of the *Ginkgo biloba* leaves powder was 3.17% (*w*/*w*).

The standards of SA (≥99.0%), PA (≥99.0%), GAA (≥99.0%) and PHBA (≥99.0%) were purchased from Shanghai Yuanye Bio-Technology Co., Ltd (Shanghai, China). The 6-HKA (≥99.8%) was donated by Zhejiang Conba Pharmaceutical Co., Ltd (Hangzhou, China). Choline chloride (≥98.0%), citric acid (≥99.5%), d-sorbitol (BR) were purchased from Sinopharm Chemical Reagent Co., Ltd (Shanghai, China). Phosphorous acid (chromatographic grade, 85–90%), dl-malic acid (≥99.0%), malonic acid (≥99.0%), levulinic acid (≥99.0%) and 2-pyrrolidinecarboxylic acid (≥99.0%) were obtained from Aladdin Chemistry Co., Ltd (Shanghai, China). Glycolic acid (≥98.0%), xylitol (≥99.0%) and betaine (≥99.0%) were acquired from Shanghai Macklin Biochemical Co., Ltd (Shanghai, China). Acetonitrile (chromatographic grade) was purchased from Tedia Company, Inc. (Shanghai, China). Deionized water was obtained by a Milli-Q water purification system (Millipore, Billerica, MA, USA). All other reagents and chemicals used in this work were of analytical reagent grade.

### 2.2. HPLC Analysis of Five Aromatic Acids

**Figure 2:**
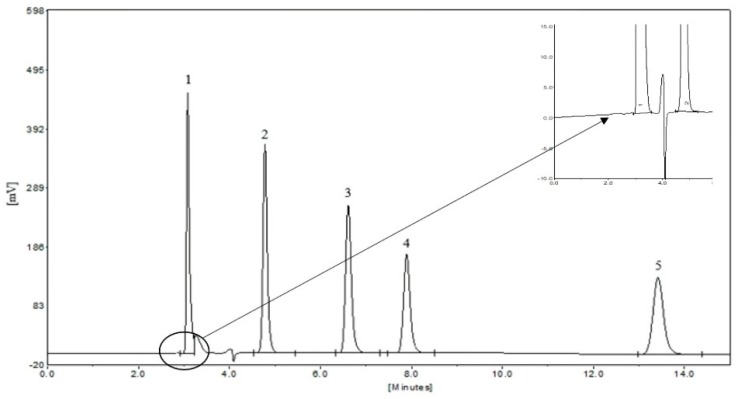
HPLC chromatogram of the five aromatic acids, peaks: (**1**) Shikimic acid (SA); (**2**) Gallic acid (GAA); (**3**) 6-hydroxykynurenic acid (6-HKA); (**4**) Protocatechuic acid (PA) and (**5**) *p*-hydroxybenzoic acid (PHBA).

HPLC analysis was performed on an Elite HPLC system (Dalian, China). The SinoChrom ODS-BP column (4.6 nm × 200 mm, 5.0 µm) and the Jiajie protection column were purchased from Elite (Dalian, China). The mobile phase was acetonitrile-0.1% phosphorous acid solution (13:87, *v*/*v*) at a flow rate of 1 mL/min. The column temperature was controlled at 30 °C. Detection was performed at a wavelength of 215 nm for 0–10 min and 256 nm for 10–15 min [9,18]. The chromatogram of the five aromatic acids was shown in Figure 2. The retention times of SA, GAA, 6-HKA, PA, and PHBA were 3.08 min, 4.78 min, 6.61 min, 7.89 min and 13.42 min, respectively. The contents of the five aromatic acids were calculated by means of five calibration curves established with five regression equations, which were listed in Table S1 in the Electronic Supporting Information.

### 2.3. DESs Preparation

In this work, 54 DESs were prepared to investigate their extraction abilities for five aromatic acids during the initial screening. According to previous work [19], the DESs were simply produced by mixing two components under a certain molar ratio and heating at 80 °C with constant stirring. The prepared DESs are listed in Table S2 in the Electronic Supporting Information.

### 2.4. Extraction of Five Aromatic Acids from Ginkgo biloba Leaves Employing DESs

An accurately weighed *Ginkgo biloba* leaves powder (80 mg) was added into a 2 mL centrifuge tube, mixed with 0.8 mL DES, and extracted in an air-bath shaker at 25 °C and 250 rpm for 5 min. In order to reduce the viscosity of DESs, 30% water (*w*/*w*) was added into the DESs during the initial screening. After extraction, the extraction solution was centrifuged for 10 min at 10,000 rpm. Then, 0.5 mL of supernatant was sampled and diluted for four-fold with water. The diluted solution was filtered through a 0.45 µm membrane filter and analyzed by HPLC. The parallel experiments were carried out three times.

### 2.5. DESs Tailoring

After initial screening, the efficient components of DESs for the extraction of five aromatic acids were analyzed. Then the DESs with high extraction yields were tailored by changing the molar ratio between component 1 and component 2. Furtherly, the efficient components obtained during initial screening were added into the optimal binary DESs to design ternary DESs.

The water content in DESs can influence their polarity and reduce their viscosity [20]. Lower viscosity facilitated the mass transfer of the aromatic acids from plant material to DESs and enhanced the extraction yields. After initial DES screening and tailoring, the efficient ternary DESs were selected as the extraction solvents. The effect of water content was investigated at 0, 10, 20, 30, 40, 50, 60, 80, and 100 (*w*/*w*, %).

### 2.6. Optimization of the Extraction Procedure for Five Aromatic Acids

#### 2.6.1. Effect of the Extraction Methods

In order to compare the extraction efficiency of DESs employing different extraction methods, heating, stirring (150 rpm), water-bath shaking (150 rpm), air-bath shaking (150 rpm) and ultrasonic (50 W) methods were investigated. Except for the extraction efficiency, the significance of heating was also investigated. 25 °C and 60 °C (the optimum temperature for aromatic acid extraction) were adopted [21]. Apart from the extraction conditions signed in the brackets were different, the other extraction parameters were kept the same: solid/solvent ratio was 1:10 (*w*/*v*, g/mL), extraction time was 5 min, and the water content was the optimal value.

#### 2.6.2. Optimization of the Extraction Conditions

First of all, the solid to solvent ratio was investigated at 1:5, 1:7.5, 1:10, 1:12.5, 1:15, and 1:20 (*w*/*v*, g/mL). Secondly, under the optimum solid to solvent ratio, the extraction temperature was investigated from 25 °C to 65 °C at an interval of 5 °C. Then, the ultrasonic power was investigated from 50 W to 300 W at an interval of 50 W. At last, the extraction time was investigated from 5 min to 35 min at an interval of 5 min under the optimized extraction conditions.

## 3. Results and Discussion

### 3.1. Various Types of DESs Preparation

Employing the hydrophilic DESs to extract the natural active compounds from plant materials have been reported by some researchers [22,23,24]. The DESs are produced by mixing two components as HBAs or HBDs that are capable of hydrogen bond interactions. The common HBAs are quaternary ammonium salts, such as choline chloride and betaine, and the HBDs are usually acids and alcohols [25,26]. According to our previous work [27], some alcohols and acids can also be mixed with each other to prepare the DESs under certain molar ratios. Therefore, 1,3-butanediol, propylene glycol or xylitol were employed as one component and some acids were employed as the other component to prepare DESs. On this occasion, it is hard to define the alcohols or acids to act as HBAs or HBDs due to the complexity of the hydrogen bond formation mechanism. The two components were thus signed as component 1 and component 2.

### 3.2. Screening and Tailoring DESs for the Simultaneous Extraction of Five Aromatic Acids

#### 3.2.1. Initial DESs Screening

Forty-five DESs produced above were initially screened for simultaneous extraction of five aromatic acids from *Ginkgo biloba* leaves. Because of the various properties of different DESs, one DES may show different extraction efficiency for the five different aromatic acids. In order to facilitate the comparison, a main indicator is essential to be chosen from the five aromatic acids during the initial simultaneous extraction screening. The extraction yield of SA was around 20 mg/g in 5 min, while those of the other four aromatic acids were less than 1 mg/g. Therefore, the extraction yield of SA was chosen as the main indicator to investigate the extraction abilities of different DESs. When the extraction yield of SA was similar, the extraction yields of GAA, PA, 6-HKA and PHBA could be the auxiliary indicators.

The initial screening results were listed in Table 1. The forty-five DESs used for initial screening are binary ones formed by mixing component 1 and component 2. As for component 1, it was clear that the xylitol-based DESs showed higher extraction yields for five aromatic acids, followed by propylene glycol-based and 1,3-butanediol-based DESs, and the betaine-based DESs showed lower extraction yields. It can be speculated that the polarity of xylitol-based DESs is similar to those of aromatic acids. In other words, xylitol was an effective component to extract aromatic acids from *Ginkgo biloba* leaves. Regarding to the component 2, acid-based DESs showed higher extraction yields than those of alcohol-based DESs. There is no doubt that the physical and chemical properties of acids are approximate to those of SA, GAA, 6-HKA, PA and PHBA, which could enhance the extraction yields. The DESs produced with DL-malic acid, citric acid, glycolic acid and malonic acid as component 2 showed higher extraction yields to the SA. It can be assumed that these four components were efficient to the SA extraction. Similarly, the effective components to GAA extraction were 1,5-pentanedioic acid, dl-malic acid, citric acid, malonic acid, glycolic acid and levulinic acid. The effective components to 6-HKA extraction were glycolic acid, lactic acid, malonic acid, levulinic acid and dl-malic acid. Glycolic acid, dl-malic acid, citric acid and xylitol were effective components for extracting PA. Apart from levulinic acid and citric acid, propylene glycol, glycerol and sorbitol showed efficient extraction abilities to PHBA.

#### 3.2.2. Tailoring DESs by Changing the Molar Ratio between Component 1 and Component 2

According to the previous work [28], as the molar amount of one component in DES mixture changes, the viscosity and surface tension of the DES will change. Lower viscosity facilitated the mass transfer of the five aromatic acids from *Ginkgo biloba* leaves to DESs and enhanced the extraction yields. Lower surface tension was beneficial to DESs soaking into plant material and enhanced the extraction yields [15]. Through the initial screening, four DESs were chosen for further tailoring by changing the molar ratio between component 1 and component 2, which were ChCl-MA2, ChCl-CA, B-MA2 and X-GA. The molar ratios varying from 3:1 to 1:5 were investigated for the fine-tuning of extraction ability.

As shown in Table 2, choline chloride and citric acid could not form stable DESs at the molar ratios of 1:4 and 1:5. As the molar number of citric acid increased, the concentration of choline chloride would decrease, which reduced the interactions between choline chloride and citric acid, thus leading to salt out. The extraction yields of five aromatic acids changed significantly along with the change of the molar ratio between component 1 and component 2. As the molar numbers of acids (dl-malic acid, citric acid and glycolic acid) increased, the extraction yields of X-GA and B-MA2 went up then went down, while the extraction yields of ChCl-CA and ChCl-MA2 were on the decline. The first increase of X-GA/B-MA2 may be explained by the decrease of the viscosity and/or surface tension with the increase of the amount of glycolic acid/dl-malic acid. However, an excess of acid in a DES would lead to the concentration decline of the other component, which weakened the interactions between component 1, component 2, and targeted aromatic acids, thus decreased the extraction efficiency. As a result, the X-GA (1:4), ChCl-MA2 (1:1), ChCl-CA (1:1) and B-MA2 (1:1) were chosen for next tailoring.

#### 3.2.3. Tailoring DESs by Forming the Ternary DESs

The ternary DESs forming with addition of glycerol to the binary DESs show lower melting points and viscosities than those of binary DESs [29]. In our previous work, ternary DESs could obviously enhance the extraction yield of the polyprenyl acetates from *Ginkgo biloba* leaves [16]. The third component adding to binary DESs may change the properties of ternary DESs and thus influence the extraction yield. In this section, based on the binary DESs (X-GA (1:4), ChCl-MA2 (1:1), ChCl-CA (1:1) and B-MA2 (1:1)), novel ternary DESs were designed in order to further enhance the extraction yield. Malic acid, citric acid, levulinic acid, lactic acid, propylene glycol, 1,5-pentanedioic acid and malonic acid, which were effective components for the extraction of targeted aromatic acids during the initial screening, were chosen as the third component to design ternary DESs. The prepared ternary DESs are listed in Tables S3–S6 in the Electronic Supporting Information.

The extraction yields of five aromatic acids employed the ternary DESs based on X-GA, ChCl-MA2, ChCl-CA and B-MA2 were listed in Table 3, Table 4, Table 5 and Table 6. Compared to the binary DES, the addition of the third component could change the extraction efficiency. The extraction yields of the tailor-made ternary DESs based on X-GA (1:4) enhanced significantly when levulinic acid, propylene glycol or 1, 5-pentanedioic acid was added as the third component. However, other ternary DESs could not lead to improved extraction yields (Table 3). When adding the third component into ChCl-MA2 (1:1), only citric acid addition with a molar ratio of ChCl-MA2-CA at 1:0.5:0.5 showed higher extraction yield than that of ChCl-MA2 (1:1) (Table 4). As for binary ChCl-CA (1:1), the prepared ternary DESs ChCl-CA-PA (1:0.25:0.75) and ChCl-CA-MA1 (1:0.75:0.25) with respective addition of 1,5-pentanedioic acid and malonic acid gave higher extraction yields (Table 5). A similar result was obtained for B-MA2 (1:1) when 1, 5-pentanedioic acid was adopted as the third component (Table 6). Analyzing the results obtained above, it could be found that the tailor-made ternary DESs with polyhydric acids (citric acid, malonic acid and 1,5-pentanedioic acid) addition led to improved extraction yields. It could be speculated that the addition of polyhydric acids enhanced the hydrogen bond interactions between DES components and the target aromatic acids, thus improved the extraction yields. The extraction yields of SA, GAA, 6-HKA, PA and PHBA extracted by X-GA-P (1:3:1) were 70.02 ± 0.23 mg/g, 262.84 ± 10.56 μg/g, 17.57 ± 0.27 μg/g, 336.88 ± 13.16 μg/g and 15.68 ± 0.51 μg/g, respectively (5-4, Table 3). The extraction yields of SA, GAA, 6-HKA, PA and PHBA extracted by X-GA-PA (1:3:1) were 66.56 ± 0.81 mg/g, 223.63 ± 7.68 μg/g, 16.69 ± 0.44 μg/g, 294.11 ± 9.39 μg/g and 22.82 ± 0.02 μg/g, respectively (6-4, Table 3). Compare the results obtained from Table 3 to Table 6, these two ternary DESs gave higher extraction yields. Finally, X-GA-P, named as NGP, tailor-made from xylitol, glycolic acid and propylene glycol at a molar ratio of 1:3:1, and X-GA-PA, named as NGG, tailor-made from xylitol, glycolic acid and 1,5-pentanedioic acid at a molar ratio of 1:3:1, were chosen for next tailoring.

#### 3.2.4. Tailoring DESs by Changing the Water Content

**Figure 3:**
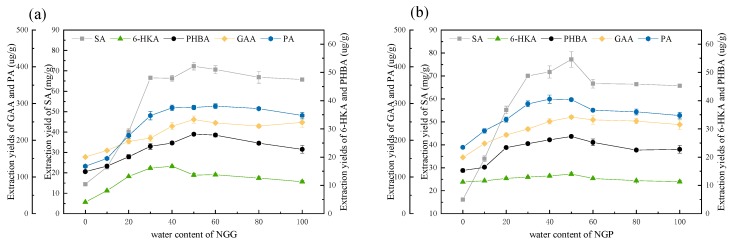
Effect of water content on the extraction yields of five aromatic acids. (**a**) Effect of water content of NGP; (**b**) Effect of water content of NGG. Extraction conditions: air-bath shaking at 250 rpm for 5 min with the solvent to solid ratio of 1:10. SA: shikimic acid, GAA: gallic acid, 6-HKA: 6-hydroxykynurenic acid, PA: protocatechuic acid, and PHBA: *p*-hydroxybenzoic acid.

It is efficient to adjust the polarity, reduce the viscosity and increase the mass transfer during the extraction process by adding a certain amount of water into DESs [30]. The two ternary DESs NGP and NGG were further tailored by changing the water content. The results were shown in Figure 3. It could be found that the fine-tuning of the water content would bring a great change in the extraction yield. For the two DESs, the extraction yields presented the same trend of increasing initially and then decreasing. When the water content was less than 50% (*w*/*w*), the extraction yields of five aromatic acids increased with the increase of water content. On one hand, the viscosity of DESs could decrease with the increase of water content [24]. Therefore, the mass transfer between DESs and *Ginkgo biloba* leaves powder would increase, which led to the increase in extraction yields. On the other hand, water addition would change the polarity of DESs. It could be inferred that the polarity of DESs was closer to those of the target five aromatic acids with the increase of water content. The extraction yields increased because any substances with the same polarity could be more likely to dissolve each other. When the water content was higher than 50% (*w*/*w*), the extraction efficiency decreased with further increase of water content. This phenomenon could be explained by the hydrogen bond weakening between the components of DESs due to the hydrogen bond strengthening between water and DESs [31]. That was to say, the hydrogen bond network of DESs were destroyed by too much water addition. When the water content was 50% (*w*/*w*), the extraction yields of five aromatic acids were the highest. Therefore, NGG with 50% water content (*w*/*w*) named as NGG50 and NGP with 50% water content (*w*/*w*) named as NGP50 were selected for the next optimization of extraction conditions.

### 3.3. Optimization of the Extraction Conditions

In order to optimize the extraction conditions, the effects of five extraction methods on the extraction yields of five aromatic acids were investigated first of all. Then, the extraction parameters such as solid to solvent ratio, extraction temperature, ultrasonic power and extraction time which might influence the extraction yields were investigated using one-variable-at-a-time method.

#### 3.3.1. Comparison of the Extraction Methods

**Figure 4:**
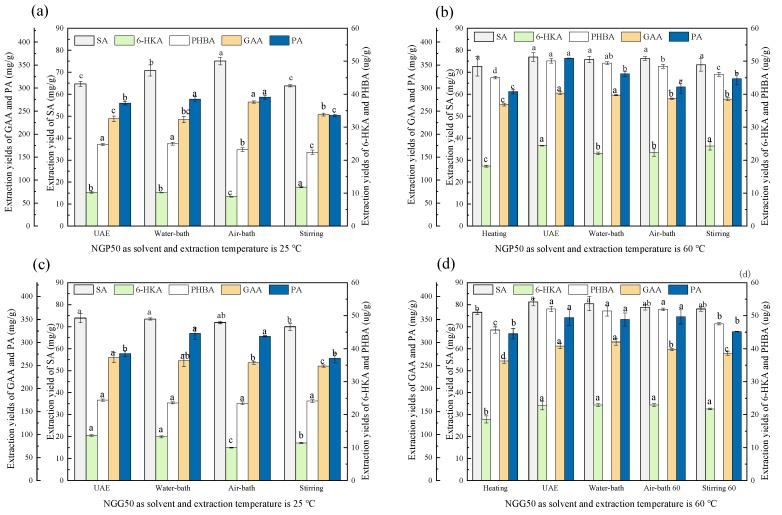
Effect of extraction methods on the extraction yields of five aromatic acid with NGP50 and NGG50 as the extraction solvents. (**a**) NGP50 as extraction solvent and extraction temperature of 25 °C, (**b**) NGP50 as extraction solvent and extraction temperature of 60 °C, (**c**) NGG50 as extraction solvent and extraction temperature of 25 °C, (**d**) NGG50 as extraction solvent and extraction temperature of 60 °C. For the same aromatic acid, values with the same letter in each column are not significantly different by least-significant difference (LSD) test. SA: shikimic acid, GAA: gallic acid, 6-HKA: 6-hydroxykynurenic acid, PA: protocatechuic acid, and PHBA: p-hydroxybenzoic acid.

An appropriate and effective extraction method could improve economic efficiency of the extraction process. Therefore, the extraction efficiency of the tailor-made DESs employing different extraction methods was investigated. As shown in Figure 4, different extraction methods exhibited discernable difference in extraction yields. When NGP50 was employed as the extraction solvent to extract at 25 °C, the extraction yield of air-bath shaking method was the highest, followed by the water-bath shaking method, and the UAE and stirring methods gave lower extraction yields. When the extraction temperature increased from 25 °C to 60 °C, the extraction yields of these four extraction methods were all higher than those at 25 °C, and the differences between extraction methods were not obvious. Thus, temperature was an important influence factor during extraction process. When NGG50 was employed as the extraction solvent to extract at 25 °C, the order of the extraction ability of the four extraction methods was UAE, water-bath shaking, air-bath shaking and stirring. When the extraction temperature elevated to 60 °C, similar results were obtained as NGP50 extracting at 60 °C. On the whole, the extraction ability of UAE method was similar to that of water-bath shaking method, and thus these two methods were employed for next investigation. Compare the extraction efficiency of NGG50 and NGP50, the NGG50 was a better extraction solvent.

#### 3.3.2. Effect of the Solid to Solvent Ratio

**Figure 5:**
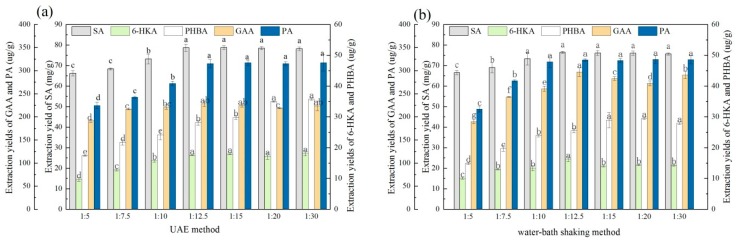
Effect of solid to solvent ratio on the amount of aromatic acids extracted from *Ginkgo biloba* leaves powder by UAE (**a**) and water-bath shaking (**b**) methods employing NGG50 as extraction solvent. For the same aromatic acid, values with the same letter in each column are not significantly different by least-significant difference (LSD) test. SA: shikimic acid, GAA: gallic acid, 6-HKA: 6-hydroxykynurenic acid, PA: protocatechuic acid, and PHBA: *p*-hydroxybenzoic acid.

A large amount of solvent could make a waste, whereas a small amount of solvent might lead to an incomplete extraction. So the effect of solid to solvent ratio on the extraction yield was investigated. As shown in Figure 5, the extraction yields of the five aromatic acids increased obviously along with the increase of the NGG50 volume before 1:12.5, and then the extraction yields were not improved with a further increase in NGG50 volume. This phenomenon indicated that the extraction yields could be improved by using a higher NGG50 volume. When the volume of NGG50 increased, the dispersion of *Ginkgo biloba* leaves powder was easier, and the mass transfer was more sufficient. When the solid to solvent ratio reached 1:12.5, the mixing of *Ginkgo biloba* leaves powder had been well-proportioned. In that case, increase in NGG50 volume could not improve the mass transfer, and thus increase the extraction yields. Through the investigation of the solid to solvent ratio, the superiority of UAE method had appeared. The extraction yield of UAE method was higher than that of water-bath shaking method at the optimized solid to solvent ratio of 1:12.5. Therefore, UAE method was selected as the extraction method to extract the five aromatic acids from *Ginkgo biloba* leaves powder.

#### 3.3.3. Effect of the Extraction Temperature

**Figure 6:**
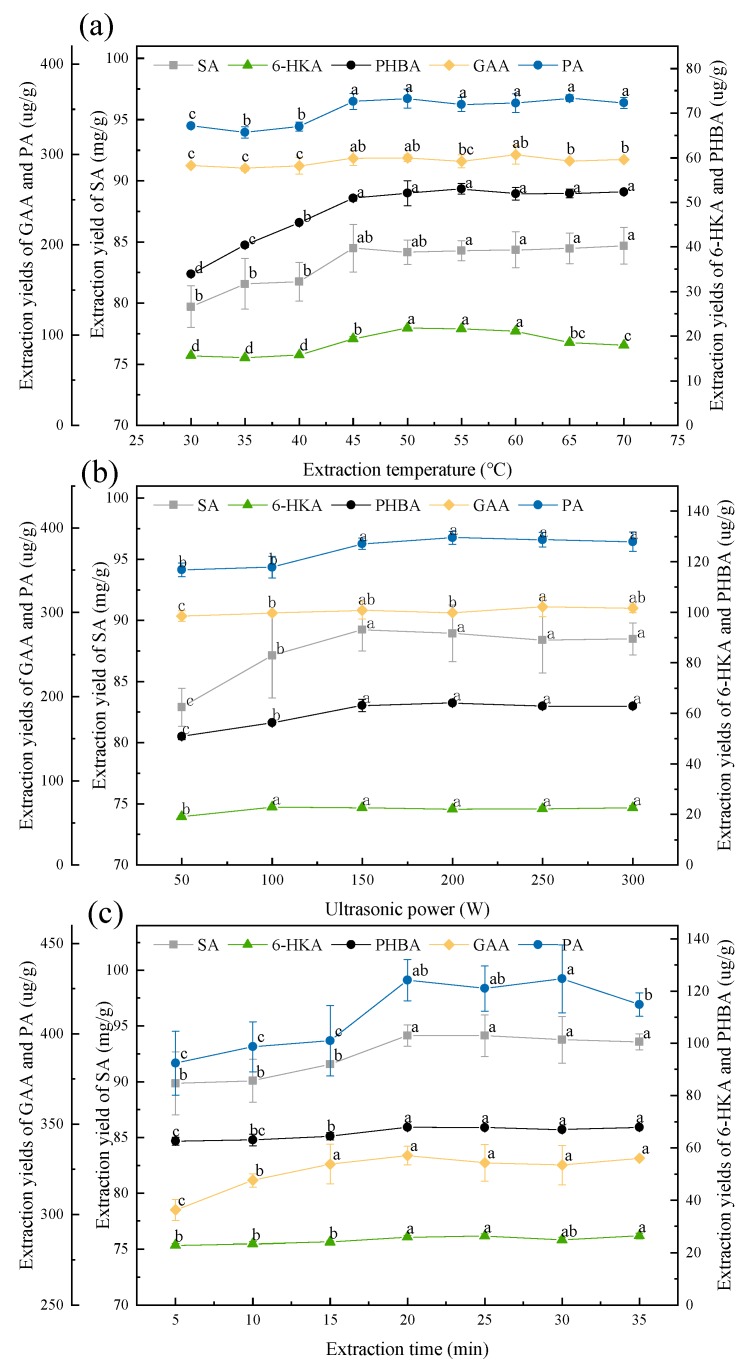
Effect of extraction parameter on the extraction yields of aromatic acids. (**a**) Effect of temperature. Extraction conditions: UAE with NGG50 as the solvent at 50 W for 5 min at the solvent to solid ratio of 1:12.5; (**b**) Effect of ultrasonic power. Extraction conditions: UAE with NGG50 as the solvent at 45 °C for 5 min at the solvent to solid ratio of 1:12.5; (**c**) Effect of extraction time. Extraction conditions: UAE with NGG50 as the solvent at 45 °C and 150 W at the solvent to solid ratio of 1:12.5. For the same aromatic acid, values with the same letter in each line are not significantly different by least-significant difference (LSD) test. SA: shikimic acid, GAA: gallic acid, 6-HKA: 6-hydroxykynurenic acid, PA: protocatechuic acid, and PHBA: *p*-hydroxybenzoic acid.

The extraction efficiency is often affected by diffusion, viscosity and solubility, which are affected by temperature [32]. At higher temperature, the solvent viscosity decreases, and the diffusivity and solubility increase, which facilitate the penetration of the target compounds from plant materials to solvent. The effect of temperature on the extraction yields of five aromatic acids was investigated from 30 °C to 70 °C. The results were shown in Figure 6a. The extraction yields of the five aromatic acids increased obviously along with the increase of the extraction temperature from 30 °C to 45 °C, and attained the maximum values at 45 °C, then kept almost unchanged. The extraction yield of GAA decreased a little when the temperature was more than 60 °C, which might result from the GAA decomposition at high temperature. 45 °C was chosen as the optimal extraction temperature.

#### 3.3.4. Effect of the Ultrasonic Power

Ultrasound can destroy the plant cell wall and release active compounds into extraction solvent by cavitation effect. Different ultrasonic power gives rise to different cavitation effect, and thus influences the extraction efficiency. Ultrasonic power of 50 to 300 W was adopted to investigate the effect on the extraction yields of five aromatic acids. Figure 6b showed the results. The extraction yields of the five aromatic acids increased along with the ultrasonic power changing from 50 to 150 W, and reached the equilibrium point at 150 W, then remained almost the same.150 W was used as the ultrasonic power to extract five aromatic acids by UAE method.

#### 3.3.5. Effect of the Extraction Time

The extension of time has a positive effect on the total extraction yield. However, too long extraction time can cause energy waste. It is necessary to seek out the proper extraction time. Extraction was performed from 5 min to 35 min. The results were shown in Figure 6c. The extraction yields of five aromatic acids were increased with the extension of time and tended to be stable after 20 min.

After the above optimization, the optimal extraction conditions were summarized as follows: the NGG50 was adopted as the extraction solvent to extract the *Ginkgo biloba* leaves powder by the UAE method at the solid to solvent ratio of 1:12.5, 45 °C and 150 W for 20 min. Under these conditions, the extraction yields of SA, GAA, 6-HKA, PA, and PHBA were 94.15 ± 0.96 mg/g, 332.69 ± 5.19 μg/g, 25.90 ± 0.61 μg/g, 429.89 ± 11.47 μg/g and 67.94 ± 0.37 μg/g, which increased by 4.78%, 38%, 14%, 12% and 8.5%, respectively, when compared with the extraction yields before optimization. In addition, the *Ginkgo biloba* leaves powder residue of first extraction was collected for second extraction under the optimal conditions. The results showed that the first extraction rates of SA, GAA, 6-HKA, PA, and PHBA were 93.69%, 82.66%, 85.55%, 84.65% and 78.41%, respectively, which demonstrated that the extraction method developed in this work could simultaneously extract the five aromatic acids from *Ginkgo biloba* leaves efficiently.

### 3.4. Evaluation of Extraction Efficiency of the NGG50 in Comparison to Conventional Solvents

**Figure 7:**
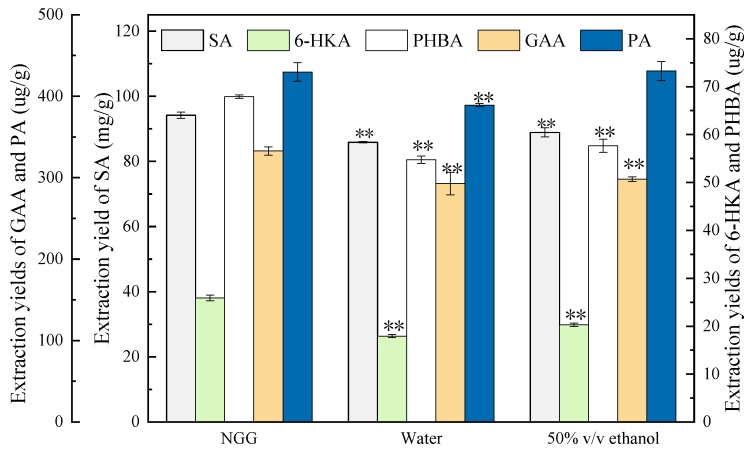
Comparison of the extraction efficiency of NGG50 with the conventional solvents under the optimized extraction conditions. ** means the significantly different by least-significant difference (LSD) test for each extraction solvent. SA: shikimic acid, GAA: gallic acid, 6-HKA: 6-hydroxykynurenic acid, PA: protocatechuic acid, and PHBA: *p*-hydroxybenzoic acid.

In order to evaluate the extraction efficiency of NGG50, the extraction yields of NGG50 and conventional solvents were compared under the same extraction conditions developed in this work. Water and 50% (*v*/*v*) ethanol were chosen as the conventional solvents according to the previous work [33]. The results were shown in Figure 7. Apart from the extraction yield of PA extracted by 50% (*v*/*v*) ethanol was close to that extracted by NGG50, the extraction yields of other four aromatic acids extracted by NGG50 were obviously higher than those extracted by water and 50% (*v*/*v*) ethanol. Usuki et al. [7] performed the extraction of SA from *Ginkgo biloba* leaves using ionic liquid (1-butyl-3-methylimidazolium chloride ([bmim]Cl)) at 150 °C, which gave an extraction yield of 2.3% (*w*/*w*). Comparatively speaking, the extraction method employing NGG50 established in this work yielded 4 times as many SA as that using [bmim]Cl at a much lower temperature, indicating that the developed method is an effective alternative. In consideration of the sustainability, biodegradability, pharmaceutical acceptable toxicity of DESs, simultaneous extraction of five aromatic acids from *Ginkgo biloba* leaves with NGG50 is a cleaner and energy-saving process for aromatic acids extraction.

## 4. Conclusions

In this work, DES-based extraction was successfully developed to simultaneously extract SA, GAA, 6-HKA, PA, and PHBA from *Ginkgo biloba* leaves. A tailor-made DES (NGG50) with the highest extraction ability for the five aromatic acids was designed by combining xylitol, glycolic acid and 1,5-pentanedioic acid at a molar ratio of 1:3:1 with 50% (*w*/*w*) water addition. Main factors affecting the NGG50-based extraction process were systematically optimized. The optimal extraction conditions were summarized as follows: the NGG50 was adopted as the extraction solvent to extract the *Ginkgo biloba* leaves powder by the UAE method at the solid to solvent ratio of 1:12.5, 45 °C and 150 W for 20 min. Under these conditions, the first extraction rates of SA, GAA, 6-HKA, PA, and PHBA was 93.69%, 82.66%, 85.55%, 84.65% and 78.41%, respectively. These results imply that NGG50 can efficiently extract more than one compound at the same time, which simplifies the technological process while guaranteeing the extraction efficiency. It will greatly reduce the energy consumption and wastes production, according to the principles of green extraction. Apart from investigating the extraction efficiency, a framework for the tailoring of DESs for higher extraction efficiency was also demonstrated in this work, which may show some references for the modulation of DESs.

In addition, the contents of five aromatic acids in *Ginkgo biloba* leaves were measured for the first time, which provide some new information for the exploitation of *Ginkgo biloba* leaves. The current resource for SA is *Illicium verum*. However, there is difficulty in supplying *Illicium verum* due to the long growth time, six years or more [34]. The content of SA in *Ginkgo biloba* leaves is about 100.49 mg/g, which is much higher than that in *Illicium verum*. *Ginkgo biloba* leaves can be used as a substitute resource for SA. For the production of 6-HKA and other aromatic acids, extracting from *Ginkgo biloba* leaves with NGG50 may be a more feasible and cleaner industrial production mode than chemical synthesis on account of low cost, sustainability, and high effectiveness.

## 5. Patents

A Chinese patent named as “A deep eutectic solvent for simultaneous extraction of five organic acids from *Ginkgo biloba* leaves: preparation and extraction” (CN 107694147A) had published.

## Figures and Tables

**Table 1:** Extraction yields (milligram of SA and micrograms of GAA, 6-HKA, PA, and PHBA per gram of *Ginkgo biloba* leaves powder) of different Deep eutectic solvents (DESs).

DESs	SA (mg/g)	GAA (µg/g)	6-HKA (µg/g)	PA (µg/g)	PHBA (µg/g)
ChCl-G	23.78 ± 0.60	224.02 ± 9.11	10.18 ± 0.21	41.08 ± 0.85	26.04 ± 0.24
ChCl-EG	25.59 ± 0.73	222.32 ± 7.21	23.73 ± 0.64	200.93 ± 2.51	17.50 ± 0.24
ChCl-P	20.45 ± 0.83	148.04 ± 4.27	21.55 ± 0.91	167.36 ± 4.84	14.17 ± 0.56
ChCl-B	16.23 ± 0.40	113.77 ± 2.38	17.83 ± 0.33	136.07 ± 3.02	19.68 ± 0.73
ChCl-DS	18.47 ± 0.81	174.81 ± 4.81	17.96 ± 0.25	162.38 ± 0.54	15.70 ± 0.03
ChCl-DG	15.77 ± 0.17	144.73 ± 2.57	6.58 ± 0.24	121.91 ± 0.23	13.42 ± 0.51
ChCl-PA	23.78 ± 0.45	411.37 ± 13.99	30.59 ± 1.08	207.94 ± 7.08	16.78 ± 0.72
ChCl-GA	25.00 ± 0.95	217.48 ± 0.54	34.39 ± 1.21	237.66 ± 4.42	16.40 ± 0.16
ChCl-MA1	25.01 ± 0.31	200.25 ± 7.76	31.90 ± 0.08	210.50 ± 4.74	16.37 ± 0.23
ChCl-MA2	42.91 ± 1.26	164.95 ± 4.92	20.71 ± 0.60	237.95 ± 7.79	16.25 ± 0.63
ChCl-LA1	23.24 ± 1.01	200.19 ± 4.82	26.65 ± 0.51	186.13 ± 3.68	40.84 ± 0.14
ChCl-LA2	20.79 ± 0.13	183.95 ± 4.25	52.64 ± 1.26	189.13 ± 6.16	16.52 ± 0.44
ChCl-CA	44.09 ± 0.23	264.49 ± 4.9	24.68 ± 0.10	187.63 ± 4.72	19.86 ± 0.21
ChCl-TA	24.26 ± 0.98	200.37 ± 0.59	27.72 ± 0.39	189.86 ± 6.34	16.14 ± 0.05
ChCl-U	26.59 ± 0.93	168.25 ± 4.41	30.74 ± 1.03	194.21 ± 8.41	20.19 ± 0.81
BE-G	17.82 ± 0.22	121.98 ± 3.42	19.51 ± 0.51	129.97 ± 6.05	13.22 ± 0.45
BE-EG	15.49 ± 0.03	150.57 ± 6.54	24.33 ± 1.06	167.68 ± 6.70	13.97 ± 0.39
BE-P	16.07 ± 0.59	502.83 ± 10.71	12.32 ± 0.55	140.56 ± 6.18	42.62 ± 0.26
BE-B	15.66 ± 0.45	120.00 ± 4.52	12.94 ± 0.46	126.19 ± 0.92	23.33 ± 1.01
BE-X	3.70 ± 0.16	209.73 ± 4.38	13.28 ± 0.28	249.72 ± 6.13	12.03 ± 0.58
BE-DS	10.55 ± 0.28	124.30 ± 3.82	17.80 ± 0.37	122.84 ± 0.62	23.86 ± 0.07
BE-GA	25.20 ± 0.45	162.56 ± 3.31	20.57 ± 0.67	156.26 ± 2.89	18.82 ± 0.53
BE-MA1	27.15 ± 1.32	248.94 ± 9.41	37.10 ± 0.84	184.09 ± 2.94	18.69 ± 0.69
BE-MA2	23.02 ± 0.80	237.65 ± 4.65	11.26 ± 0.50	183.55 ± 2.79	20.72 ± 0.72
BE-LA1	19.96 ± 0.53	156.53 ± 3.81	47.38 ± 1.84	184.09 ± 2.94	38.61 ± 1.32
BE-LA2	7.61 ± 0.09	141.97 ± 0.54	51.69 ± 2.35	166.92 ± 5.46	14.21 ± 0.19
BE-CA	39.04 ± 0.37	233.34 ± 5.22	19.75 ± 0.44	203.24 ± 3.74	16.39 ± 0.16
B-LA2	28.87 ± 0.51	188.89 ± 7.26	72.39 ± 0.90	211.55 ± 8.03	14.97 ± 0.32
B-LA1	27.81 ± 0.39	257.62 ± 2.23	23.61 ± 0.56	208.20 ± 10.33	29.59 ± 0.75
B-MA2	41.58 ± 0.55	233.31 ± 11.17	43.83 ± 2.03	221.45 ± 6.36	15.56 ± 0.30
B-CA	31.07 ± 0.16	183.33 ± 6.57	29.49 ± 1.09	272.37 ± 7.44	47.07 ± 1.23
B-GA	34.60 ± 1.38	236.58 ± 9.90	29.59 ± 1.13	218.16 ± 2.58	15.14 ± 0.42
B-PA	23.63 ± 0.78	155.78 ± 5.15	22.34 ± 0.59	200.25 ± 4.94	12.91 ± 0.50
P-LA2	29.64 ± 0.35	226.56 ± 2.22	65.38 ± 1.53	237.44 ± 5.73	13.61 ± 0.65
P-LA1	25.87 ± 0.77	177.59 ± 8.57	22.19 ± 0.95	209.62 ± 7.15	34.27 ± 0.90
P-MA2	37.32 ± 1.41	230.85 ± 8.40	20.24 ± 0.62	254.80 ± 12.37	15.09 ± 0.09
P-CA	18.85 ± 0.50	166.23 ± 7.15	27.63 ± 0.16	192.01 ± 8.48	12.80 ± 0.44
P-GA	35.00 ± 1.14	133.55 ± 2.26	30.83 ± 0.10	253.69 ± 11.14	13.07 ± 0.48
P-PA	24.12 ± 0.17	132.11 ± 5.57	23.45 ± 0.92	204.87 ± 2.72	12.17 ± 0.29
X-LA2	33.12 ± 0.82	242.25 ± 6.62	45.31 ± 1.72	192.03 ± 3.36	21.70 ± 0.70
X-LA1	32.21 ± 1.17	433.09 ± 2.49	24.44 ± 0.38	204.64 ± 8.49	22.33 ± 0.90
X-MA2	32.13 ± 0.93	276.84 ± 13.31	16.86 ± 0.22	170.61 ± 2.04	20.98 ± 0.62
X-CA	26.83 ± 0.99	123.49 ± 3.10	20.62 ± 0.46	161.19 ± 1.70	19.54 ± 0.62
X-GA	47.98 ± 0.45	305.42 ± 0.04	19.00 ± 0.56	143.14 ± 7.11	12.57 ± 0.51
X-PA	28.42 ± 0.53	169.53 ± 5.65	19.46 ± 0.90	177.33 ± 4.10	14.07 ± 0.58

The acronyms used in the first column are the names of different DESs whose composition and molar ratio are shown in Table S2 in the Electronic Supporting Information. SA: shikimic acid, GAA: gallic acid, 6-HKA: 6-hydroxykynurenic acid, PA: protocatechuic acid, and PHBA: *p*-hydroxybenzoic acid.

**Table 2:** Extraction yields (milligram of SA and micrograms of GAA, 6-HKA, PA, and PHBA per gram of *Ginkgo biloba* leaves powder) using different Deep eutectic solvents (DESs, X-GA, ChCl-MA2, ChCl-CA, and B-MA2) with different component molar ratios.

DESs	Molar Ratio	SA (mg/g)	GAA (μg/g)	6-HKA (μg/g)	PA (μg/g)	PHBA (μg/g)
X-GA	3:1	25.23 ± 0.46	201.56 ± 1.65	3.69 ± 0.08	57.81 ± 2.05	13.34 ± 0.53
	2:1	28.61 ± 1.37	204.45 ± 7.94	9.38 ± 0.31	107.74 ± 3.15	11.38 ± 0.32
	1:1	47.98 ± 0.01	305.42 ± 0.04	19.00 ± 0.56	143.14 ± 7.11	12.57 ± 0.51
	1:2	50.96 ± 0.94	248.04 ± 9.30	17.74 ± 0.64	233.07 ± 5.00	12.62 ± 0.15
	1:3	58.35 ± 1.50	281.54 ± 8.75	23.10 ± 0.48	274.20 ± 8.43	13.68 ± 0.43
	1:4	58.80 ± 0.68	363.69 ± 4.14	47.04 ± 1.54	301.51 ± 4.40	15.26 ± 0.20
	1:5	51.76 ± 0.68	300.39 ± 10.22	32.00 ± 0.35	280.86 ± 3.13	14.81 ± 0.20
ChCl-MA2	1:1	42.91 ± 1.26	164.95 ± 4.92	20.71 ± 0.60	237.95 ± 7.79	16.25 ± 0.63
	1:2	39.64 ± 1.27	154.64 ± 4.33	12.86 ± 0.46	222.09 ± 10.05	18.66 ± 0.16
	1:3	37.70 ± 0.49	166.37 ± 5.76	18.99 ± 0.21	167.86 ± 0.08	22.29 ± 0.92
	1:4	37.09 ± 0.22	107.98 ± 3.73	18.91 ± 0.19	216.10 ± 1.79	18.94 ± 0.85
	1:5	28.71 ± 0.69	80.70 ± 1.44	18.38 ± 0.69	214.53 ± 7.14	18.94 ± 0.19
ChCl-CA	1:1	44.09 ± 0.23	264.49 ± 4.90	24.68 ± 0.1	187.63 ± 4.72	19.86 ± 0.21
	1:2	30.10 ± 1.48	326.94 ± 4.88	14.07 ± 0.33	131.62 ± 1.90	14.43 ± 0.43
	1:3	26.14 ± 0.13	159.49 ± 4.35	13.35 ± 0.43	119.76 ± 1.31	14.02 ± 0.19
	1:4 ^a^	-	-	-	-	-
	1:5 ^b^	-	-	-	-	-
B-MA2	3:1	28.15 ± 0.47	50.60 ± 2.02	14.44 ± 0.31	122.86 ± 2.12	18.40 ± 0.44
	2:1	32.16 ± 1.53	153.18 ± 3.33	13.87 ± 0.53	133.69 ± 6.42	18.93 ± 0.89
	1:1	41.58 ± 0.55	233.31 ± 11.17	43.83 ± 2.03	221.45 ± 6.36	15.56 ± 0.30
	1:2	40.84 ± 1.71	321.20 ± 2.45	16.81 ± 0.21	161.22 ± 0.38	21.68 ± 0.86
	1:3	40.67 ± 0.42	431.65 ± 8.36	35.72 ± 0.02	177.93 ± 5.26	22.42 ± 0.81
	1:4	34.07 ± 0.55	520.44 ± 4.63	17.88 ± 0.77	168.12 ± 4.82	18.33 ± 0.64
	1:5	30.61 ± 0.89	273.85 ± 2.80	12.68 ± 1.20	168.28 ± 1.76	18.45 ± 0.37

The acronyms used in the first column are the names of different DESs whose composition and molar ratio are shown in Table S2 in the Electronic Supporting Information. “a” and “b” show that the choline chloride and citric acid could not form stable DESs at the selected molar ratios. SA: shikimic acid, GAA: gallic acid, 6-HKA: 6-hydroxykynurenic acid, PA: protocatechuic acid, and PHBA: *p*-hydroxybenzoic acid.

**Table 3:** Extraction yields (milligram of SA and micrograms of GAA, 6-HKA, PA, and PHBA per gram of *Ginkgo biloba* leaves powder) of the ternary Deep eutectic solvents (DESs) based on X-GA.

DESs	SA (mg/g)	GAA (μg/g)	6-HKA (μg/g)	PA (μg/g)	PHBA (μg/g)
1-1	37.14 ± 1.66	149.36 ± 2.29	11.51 ± 1.10	256.7 ± 12.5	24.82 ± 0.30
1-2	44.24 ± 0.24	87.63 ± 2.08	12.71 ± 1.02	282.47 ± 4.60	28.28 ± 0.40
1-3	49.64 ± 1.83	94.01 ± 0.93	11.50 ± 0.45	265.13 ± 11.3	17.38 ± 0.01
1-4	48.93 ± 1.83	182.82 ± 0.42	18.94 ± 0.78	301.24 ± 8.19	14.14 ± 0.27
1-5	58.80 ± 0.67	163.69 ± 4.14	17.04 ± 1.54	301.51 ± 4.40	15.26 ± 0.20
2-1 ^a^	-	-	-	-	-
2-2	35.59 ± 1.01	193.61 ± 3.14	17.76 ± 0.07	204.18 ± 7.23	13.63 ± 0.56
2-3	50.05 ± 0.15	123.51 ± 1.15	14.72 ± 0.90	260.18 ± 0.43	12.87 ± 0.17
2-4	52.56 ± 1.62	185.54 ± 3.31	12.01 ± 0.17	287.08 ± 12.6	14.55 ± 0.48
2-5	58.80 ± 0.67	163.69 ± 4.14	17.04 ± 1.54	301.51 ± 4.40	15.26 ± 0.20
3-1	25.11 ± 0.86	137.59 ± 11.4	13.05 ± 0.47	191.55 ± 7.01	15.16 ± 0.36
3-2	52.23 ± 1.49	143.98 ± 8.22	18.01 ± 0.64	251.09 ± 5.08	17.07 ± 0.47
3-3	57.59 ± 0.90	158.40 ± 1.48	14.01 ± 0.01	266.67 ± 12.2	17.04 ± 0.40
3-4	61.67 ± 2.31	157.06 ± 0.68	15.31 ± 0.90	282.55 ± 4.66	15.26 ± 0.42
3-5	58.80 ± 0.67	133.69 ± 4.14	17.04 ± 1.54	301.51 ± 4.40	15.26 ± 0.20
4-1	40.13 ± 1.82	169.95 ± 7.12	17.18 ± 1.29	280.20 ± 6.56	14.69 ± 0.70
4-2	47.88 ± 0.98	123.16 ± 14.5	10.37 ± 1.48	285.30 ± 4.24	14.89 ± 0.51
4-3	51.33 ± 1.46	168.29 ± 10.9	17.26 ± 0.95	272.23 ± 8.87	15.11 ± 0.36
4-4	50.05 ± 0.54	143.76 ± 1.02	16.77 ± 0.58	254.08 ± 7.88	15.05 ± 0.21
4-5	58.80 ± 0.67	163.69 ± 4.14	17.04 ± 1.54	301.51 ± 4.40	15.26 ± 0.20
5-1	27.37 ± 0.73	147.22 ± 5.76	19.19 ± 0.01	175.62 ± 7.75	13.61 ± 0.30
5-2	35.22 ± 1.75	187.14 ± 6.95	11.10 ± 0.57	204.07 ± 1.90	13.96 ± 0.22
5-3	41.10 ± 1.73	183.06 ± 6.53	19.64 ± 0.57	227.41 ± 10.1	12.59 ± 0.52
5-4	70.02 ± 0.23	162.84 ± 10.6	17.57 ± 0.27	336.88 ± 13.2	15.68 ± 0.51
5-5	58.80 ± 0.67	163.69 ± 4.14	17.04 ± 1.54	301.51 ± 4.40	15.26 ± 0.20
6-1 ^a^	-	-	-	-	-
6-2 ^a^	-	-	-	-	-
6-3 ^a^	-	-	-	-	-
6-4	66.56 ± 0.81	123.63 ± 7.68	16.69 ± 0.44	294.11 ± 9.39	22.82 ± 0.02
6-5	58.80 ± 0.67	163.69 ± 4.14	17.04 ± 1.54	301.51 ± 4.40	15.26 ± 0.20
7-1 ^a^	-	-	-	-	-
7-2 ^a^	-	-	-	-	-
7-3	56.03 ± 2.45	106.88 ± 3.52	14.41 ± 0.6	303.46 ± 35.2	20.84 ± 0.89
7-4	56.50 ± 2.38	131.67 ± 14.1	14.50 ± 1.90	315.80 ± 3.80	16.60 ± 0.30
7-5	58.80 ± 0.67	163.69 ± 4.14	17.04 ± 1.54	301.51 ± 4.40	15.26 ± 0.20

The acronyms used in the first column are the names of different DESs whose composition and molar ratio are shown in Table S3 in the Electronic Supporting Information. “a” shows that the third component addition could not form stable DESs at selected molar ratios. SA: shikimic acid, GAA: gallic acid, 6-HKA: 6-hydroxykynurenic acid, PA: protocatechuic acid, and PHBA: *p*-hydroxybenzoic acid.

**Table 4:** Extraction yields (milligram of SA and micrograms of GAA, 6-HKA, PA, and PHBA per gram of *Ginkgo biloba* leaves powder) of the ternary Deep eutectic solvents (DESs) based on ChCl-MA2.

DESs	SA (mg/g)	GAA (μg/g)	6-HKA (μg/g)	PA (μg/g)	PHBA (μg/g)
8-1	31.18 ± 0.20	164.49 ± 4.90	14.68 ± 0.10	187.63 ± 4.72	19.86 ± 0.21
8-2	36.67 ± 1.17	184.26 ± 3.19	13.25 ± 0.22	151.47 ± 3.81	16.67 ± 0.38
8-3	46.98 ± 0.05	128.85 ± 2.37	10.93 ± 0.20	194.70 ± 3.24	21.94 ± 0.68
8-4	42.52 ± 0.08	137.57 ± 5.43	10.84 ± 0.21	212.63 ± 4.87	19.90 ± 0.14
8-5	42.91 ± 1.26	164.95 ± 4.92	10.71 ± 0.60	237.95 ± 7.79	16.25 ± 0.63
9-1	23.24 ± 1.01	100.19 ± 4.82	16.65 ± 0.51	186.13 ± 3.68	40.84 ± 0.14
9-2	35.03 ± 0.89	126.47 ± 4.60	13.36 ± 0.09	151.12 ± 5.97	11.02 ± 0.31
9-3	37.68 ± 0.06	155.88 ± 6.06	15.53 ± 0.46	162.01 ± 4.74	10.96 ± 0.09
9-4	40.01 ± 0.30	162.88 ± 4.37	18.74 ± 0.74	139.58 ± 0.82	11.03 ± 0.34
9-5	42.91 ± 1.26	164.95 ± 4.92	11.59 ± 0.50	237.95 ± 7.79	16.25 ± 0.63
10-1	20.79 ± 0.13	183.95 ± 4.25	12.64 ± 0.84	189.13 ± 6.16	16.52 ± 0.44
10-2	35.53 ± 0.49	124.28 ± 2.19	12.86 ± 0.48	175.35 ± 7.10	11.43 ± 0.16
10-3	36.49 ± 0.61	138.05 ± 6.57	19.34 ± 0.14	154.73 ± 1.90	16.07 ± 0.75
10-4	36.72 ± 0.65	102.49 ± 4.53	13.87 ± 0.27	153.54 ± 1.38	11.11 ± 0.23
10-5	42.91 ± 1.26	164.95 ± 4.92	20.71 ± 0.60	237.95 ± 7.79	16.25 ± 0.63
11-1 ^a^	-	-	-	-	-
11-2	18.29 ± 0.59	97.71 ± 2.19	7.64 ± 0.04	118.15 ± 4.03	11.32 ± 0.41
11-3	22.94 ± 0.80	139.39 ± 2.68	8.52 ± 0.19	158.79 ± 3.84	11.72 ± 0.40
11-4	27.34 ± 0.51	144.88 ± 5.80	9.62 ± 0.16	173.17 ± 4.44	11.07 ± 0.31
11-5	42.91 ± 1.26	164.95 ± 4.92	10.71 ± 0.60	237.95 ± 7.79	16.25 ± 0.63
12-1	23.78 ± 0.45	111.37 ± 14.0	10.59 ± 1.08	207.94 ± 7.08	16.78 ± 0.72
12-2	27.96 ± 0.93	185.20 ± 8.79	10.00 ± 0.09	157.90 ± 4.15	10.39 ± 0.40
12-3	34.47 ± 1.13	185.72 ± 8.75	13.29 ± 0.25	176.67 ± 5.07	12.98 ± 0.23
12-4	35.70 ± 0.77	146.81 ± 0.98	13.00 ± 0.35	185.11 ± 5.66	11.96 ± 0.14
12-5	42.91 ± 1.26	164.95 ± 4.92	10.71 ± 0.60	237.95 ± 7.79	16.25 ± 0.63
13-1	25.01 ± 0.31	100.25 ± 7.76	11.90 ± 0.08	210.50 ± 4.74	16.37 ± 0.23
13-2	31.42 ± 0.54	119.96 ± 4.20	12.37 ± 0.07	206.66 ± 4.88	14.85 ± 0.14
13-3	38.97 ± 1.36	167.08 ± 4.03	15.88 ± 0.07	243.57 ± 10.9	12.19 ± 0.40
13-4	39.40 ± 1.78	180.78 ± 6.68	14.05 ± 0.18	210.78 ± 4.70	11.64 ± 0.34
13-5	42.91 ± 1.26	164.95 ± 4.92	20.71 ± 0.60	237.95 ± 7.79	16.25 ± 0.63

The acronyms used in the first column are the names of different DESs whose composition and molar ratio are shown in Table S4 in the Electronic Supporting Information. “a” shows that the third component addition could not form stable DES at selected molar ratio. SA: shikimic acid, GAA: gallic acid, 6-HKA: 6-hydroxykynurenic acid, PA: protocatechuic acid, and PHBA: *p*-hydroxybenzoic acid.

**Table 5:** Extraction yields (milligram of SA and micrograms of GAA, 6-HKA, PA, and PHBA per gram of *Ginkgo biloba* leaves powder) of the ternary Deep eutectic solvents (DESs) based on ChCl-CA.

DESs	SA (mg/g)	GAA (μg/g)	6-HKA (μg/g)	PA (μg/g)	PHBA (μg/g)
14-1	23.24 ± 1.01	200.19 ± 4.82	16.65 ± 0.51	186.13 ± 3.68	40.84 ± 0.14
14-2	28.37 ± 1.29	180.57 ± 1.99	10.62 ± 0.31	153.67 ± 5.90	21.23 ± 0.22
14-3	30.84 ± 0.65	165.97 ± 5.58	10.95 ± 0.13	204.52 ± 8.48	11.93 ± 0.23
14-4	29.78 ± 1.04	180.00 ± 2.80	10.78 ± 0.19	198.21 ± 7.99	11.27 ± 0.30
14-5	44.09 ± 0.23	264.49 ± 4.90	14.68 ± 0.10	187.63 ± 4.72	19.86 ± 0.21
15-1	20.79 ± 0.13	183.95 ± 4.25	12.64 ± 0.84	189.13 ± 6.16	16.52 ± 0.44
15-2	25.76 ± 1.07	174.46 ± 0.81	14.31 ± 0.13	178.25 ± 6.95	20.49 ± 0.78
15-3	25.70 ± 0.86	241.97 ± 10.6	16.49 ± 0.28	202.97 ± 2.51	18.78 ± 0.84
15-4	37.19 ± 0.91	211.99 ± 0.55	19.75 ± 0.05	242.74 ± 4.86	21.41 ± 0.40
15-5	44.09 ± 0.23	264.49 ± 4.90	14.68 ± 0.10	187.63 ± 4.72	19.86 ± 0.21
16-1 ^a^	-	-	-	-	-
16-2	20.87 ± 0.44	219.76 ± 11.0	16.01 ± 0.27	158.10 ± 4.98	17.84 ± 0.73
16-3	29.58 ± 0.33	260.91 ± 8.78	13.85 ± 0.42	174.52 ± 4.90	19.38 ± 0.84
16-4	39.25 ± 1.04	280.17 ± 2.16	11.28 ± 0.05	184.27 ± 5.79	21.04 ± 0.66
16-5	44.09 ± 0.23	264.49 ± 4.90	14.68 ± 0.10	187.63 ± 4.72	19.86 ± 0.21
17-1	35.67 ± 0.67	111.37 ± 14.0	19.73 ± 0.35	207.94 ± 7.08	16.78 ± 0.72
17-2	49.11 ± 1.57	264.16 ± 3.18	12.93 ± 1.03	297.12 ± 8.03	18.57 ± 0.34
17-3	41.32 ± 1.18	213.04 ± 4.50	19.7 ± 0.91	268.72 ± 5.99	19.42 ± 0.74
17-4	40.74 ± 2.00	209.41 ± 4.71	12.91 ± 0.60	216.47 ± 4.44	16.93 ± 0.65
17-5	44.09 ± 0.23	264.49 ± 4.90	14.68 ± 0.10	187.63 ± 4.72	19.86 ± 0.21
18-1	37.51 ± 0.47	200.25 ± 7.76	18.21 ± 1.07	210.5 ± 4.74	16.37 ± 0.23
18-2	42.21 ± 0.30	251.42 ± 4.20	15.74 ± 1.19	222.09 ± 3.90	18.04 ± 0.80
18-3	42.79 ± 0.55	242.45 ± 0.64	13.34 ± 0.40	272.46 ± 1.11	20.79 ± 0.90
18-4	45.49 ± 0.18	201.05 ± 3.66	10.39 ± 0.49	254.53 ± 5.15	18.15 ± 0.38
18-5	44.09 ± 0.23	264.49 ± 4.90	14.68 ± 0.10	187.63 ± 4.72	19.86 ± 0.21

The acronyms used in the first column are the names of different DESs whose composition and molar ratio are shown in Table S5 in the Electronic Supporting Information. “a” shows that the third component addition could not form stable DES at selected molar ratio. SA: shikimic acid, GAA: gallic acid, 6-HKA: 6-hydroxykynurenic acid, PA: protocatechuic acid, and PHBA: *p*-hydroxybenzoic acid.

**Table 6:** Extraction yields (milligram of SA and micrograms of GAA, 6-HKA, PA, and PHBA per gram of *Ginkgo biloba* leaves powder) of the ternary Deep eutectic solvents (DESs) based on B-MA2.

DESs	SA (mg/g)	GAA (μg/g)	6-HKA (μg/g)	PA (μg/g)	PHBA (μg/g)
19-1	31.07 ± 0.16	166.23 ± 7.15	9.49 ± 0.73	272.37 ± 7.44	47.07 ± 1.23
19-2	27.79 ± 0.40	214.74 ± 10.4	18.87 ± 0.40	241.08 ± 6.21	16.07 ± 0.40
19-3	25.61 ± 0.25	208.26 ± 3.68	12.02 ± 0.23	301.09 ± 2.60	14.18 ± 0.54
19-4	31.17 ± 0.15	229.53 ± 9.45	16.61 ± 0.19	272.19 ± 4.35	15.11 ± 0.30
19-5	41.58 ± 0.55	233.31 ± 11.2	13.83 ± 1.35	221.45 ± 6.36	15.56 ± 0.30
20-1	27.81 ± 0.39	257.62 ± 2.23	13.61 ± 0.56	208.20 ± 10.3	29.59 ± 0.75
20-2	27.06 ± 0.67	88.82 ± 3.04	17.92 ± 0.58	172.45 ± 6.10	11.02 ± 0.37
20-3	28.20 ± 0.45	169.89 ± 6.22	18.14 ± 0.18	172.04 ± 3.41	12.10 ± 0.03
20-4	34.87 ± 0.67	271.81 ± 6.28	16.95 ± 0.09	217.78 ± 3.02	11.12 ± 0.54
20-5	41.58 ± 0.55	233.31 ± 11.2	13.83 ± 1.35	221.45 ± 6.36	15.56 ± 0.30
21-1	28.87 ± 0.51	188.89 ± 7.26	12.39 ± 0.90	211.55 ± 8.03	14.97 ± 0.32
21-2	26.52 ± 1.15	80.68 ± 2.57	18.77 ± 0.82	208.79 ± 4.40	12.32 ± 0.10
21-3	33.70 ± 0.24	100.43 ± 3.82	14.87 ± 0.76	197.62 ± 1.71	11.01 ± 0.19
21-4	35.75 ± 0.32	155.04 ± 5.62	10.56 ± 1.25	229.50 ± 10.4	11.59 ± 0.31
21-5	41.58 ± 0.55	233.31 ± 11.2	13.83 ± 1.35	221.45 ± 6.36	15.56 ± 0.30
22-1 ^a^	-	-	-	-	-
22-2	22.64 ± 0.81	112.94 ± 1.11	10.69 ± 0.25	160.06 ± 6.84	11.76 ± 0.24
22-3	26.93 ± 0.37	178.24 ± 4.13	11.38 ± 0.87	185.78 ± 0.72	11.22 ± 0.10
22-4	28.97 ± 1.04	94.25 ± 1.87	15.32 ± 0.88	217.78 ± 8.11	14.08 ± 0.25
22-5	41.58 ± 0.55	233.31 ± 11.2	13.83 ± 1.35	221.45 ± 6.36	15.56 ± 0.30
23-1 ^a^	-	-	-	-	-
23-2	51.56 ± 0.34	141.68 ± 2.55	15.98 ± 1.06	275.93 ± 2.72	15.50 ± 0.15
23-3	54.25 ± 1.26	259.33 ± 8.62	10.87 ± 0.60	286.31 ± 4.27	16.50 ± 0.33
23-4	47.55 ± 1.78	242.84 ± 9.34	16.35 ± 1.14	204.45 ± 2.18	15.12 ± 0.49
23-5	41.58 ± 0.55	233.31 ± 11.2	13.83 ± 1.35	221.45 ± 6.36	15.56 ± 0.30
24-1	41.73 ± 0.37	190.98 ± 4.33	16.70 ± 0.85	269.59 ± 10.1	14.60 ± 0.40
24-2	40.38 ± 0.82	186.48 ± 7.46	10.00 ± 0.56	299.50 ± 1.04	15.80 ± 0.37
24-3	31.72 ± 0.20	216.30 ± 3.62	12.88 ± 1.21	330.73 ± 15.7	16.73 ± 0.54
24-4	34.02 ± 0.46	232.19 ± 3.71	10.62 ± 0.89	273.51 ± 7.66	15.49 ± 0.24
24-5	41.58 ± 0.55	233.31 ± 11.2	13.83 ± 1.35	221.45 ± 6.36	15.56 ± 0.30

The acronyms used in the first column are the names of different DESs whose composition and molar ratio are shown in Table S6 in the Electronic Supporting Information. “a” shows that the third component addition could not form stable DESs at selected molar ratios. SA: shikimic acid, GAA: gallic acid, 6-HKA: 6-hydroxykynurenic acid, PA: protocatechuic acid, and PHBA: *p*-hydroxybenzoic acid.

## Supplementary Materials

The following are available online, Table S1: Standard curves of five aromatic acids, Table S2: List of the prepared binary DESs for initial screening, Table S3: List of the prepared ternary DESs based on X-GA, Table S4: List of the prepared ternary DESs based on ChCl-MA2, Table S5: List of the prepared ternary DESs based on ChCl-CA, Table S6: List of the prepared ternary DESs based on B-MA2.
