# Supplementary material for: Tailor-Made Deep Eutectic Solvents for Simultaneous Extraction of Five Aromatic Acids from Ginkgo biloba Leaves
Source: Molecules. 2018 Dec 5;23(12):3214. doi: 10.3390/molecules23123214 (PMC6321571; doi:10.3390/molecules23123214)
Supplement: Supplementary file 1 [file molecules-23-03214-s001.pdf]

*molecules*

# Tailor-made Deep Eutectic Solvents for Simultaneous Extraction of Five Aromatic Acids from *Ginkgo biloba* Leaves

**Jun Cao [1,4], Huimin Wang [1], Wei Zhang [1], Fuliang Cao [4], Geli Ma [2], Erzheng Su [1,3,4,*]**

1 Department of Food Science and Technology, College of Light Industry and Food Engineering, Nanjing Forestry University, Nanjing 210037, China; aizhujj@qq.com

2 School of Food and Biological Engineering, Zhengzhou University of Light Industry, Zhengzhou 450002, China; mageli@zzuli.edu.cn

3 State Key Laboratory of Natural Medicines, China Pharmaceutical University, Nanjing 210009, China; ezhsu@njfu.edu.cn

4 Co-innovation Center for the Sustainable Forestry in Southern China, College of Forestry, Nanjing Forestry University, Nanjing 210037, China; 13913948118@163.com

* Correspondence: ezhsu@njfu.edu.cn; Tel.: +86-25-8542-8845

## 1. Supplementary tables

**Tabel S1.** Standard curves of five aromatic acids.

| | Regression Equation | $R^2$ | Concentration Range (μg/mL) | LOD (ug/mL) | LOQ (ug/mL) |
|---|---|---|---|---|---|
| SA | y = 917.63x + 21.489 | 0.9993 | 203–2030 | 26.57 | 80.51 |
| GA | y = 10006x − 1.1782 | 0.9996 | 200–200 | 0.16 | 0.49 |
| 6-HKA | y = 4.9038x − 0.2523 | 0.9997 | 14–140 | 0.72 | 2.19 |
| PA | y = 3.1713x − 10.06 | 0.9993 | 22–220 | 2.03 | 6.17 |
| PHBA | y = 4.9761x − 10.141 | 0.9993 | 16–160 | 0.49 | 1.49 |

**Table S2.** List of the prepared binary DESs for initial screening.

| Abbreviation | Component 1 | Component 2 | Molar Ratio | Appearance at 45 °C |
|---|---|---|---|---|
| ChCl-G | Choline chloride | Glycerol | 1:2 | Transparent liquid |
| ChCl-EG | Choline chloride | Ethylene glycol | 1:2 | Transparent liquid |
| ChCl-P | Choline chloride | Propylene glycol | 1:2 | Transparent liquid |
| ChCl-B | Choline chloride | 1,3-Butanediol | 1:3 | Transparent liquid |
| ChCl-DS | Choline chloride | D-Sorbitol | 1:1 | Transparent viscous liquid |
| ChCl-DG | Choline chloride | D-(+)-Glucose | 1:1 | Transparent viscous liquid |
| ChCl-PA | Choline chloride | 1,5-Pentanedioic acid | 1:1 | Transparent liquid |
| ChCl-GA | Choline chloride | Glycolic acid | 1:1 | Transparent liquid |
| ChCl-MA1 | Choline chloride | Malonic acid | 1:1 | Transparent liquid |
| ChCl-MA2 | Choline chloride | DL-Malic acid | 1:1 | Transparent viscous liquid |
| ChCl-LA1 | Choline chloride | Levulinic acid | 1:2 | Transparent liquid |
| ChCl-LA2 | Choline chloride | Lactic acid | 1:1 | Transparent liquid |
| ChCl-CA | Choline chloride | Citric acid | 1:1 | Transparent viscous liquid |
| ChCl-TA | Choline chloride | L(+)-Tartaric acid | 2:1 | Transparent viscous liquid |
| ChCl-U | Choline chloride | Urea | 1:2 | Transparent liquid |
| BE-G | Betaine | Glycerol | 1:2 | Transparent liquid |
| BE-EG | Betaine | Ethylene glycol | 1:3 | Transparent liquid |
| BE-P | Betaine | Propylene glycol | 1:3 | Transparent liquid |
| BE-B | Betaine | 1,3-Butanediol | 1:3 | Transparent liquid |
| BE-X | Betaine | Xylitol | 1:2 | Transparent viscous liquid |
| BE-DS | Betaine | D-Sorbitol | 1:2 | Transparent viscous liquid |
| BE-GA | Betaine | Glycolic acid | 1:1 | Transparent liquid |
| BE-MA1 | Betaine | Malonic acid | 1:2 | Transparent viscous liquid |
| BE-OA | Betaine | Oxalic acid | 1:2 | Failure of preparation |
| BE-MA2 | Betaine | DL-Malic acid | 1:1 | Transparent viscous liquid |
| BE-LA1 | Betaine | Levulinic acid | 1:2 | Transparent liquid |
| BE-LA2 | Betaine | Lactic acid | 1:1 | Transparent liquid |
| BE-CA | Betaine | Citric acid | 1:1 | Transparent viscous liquid |
| BE-TA | Betaine | L(+)-Tartaric acid | 2:1 | Failure of preparation |
| PR-G | 2-Pyrrolidinecarboxylic acid | Glycerol | 1:1 | Failure of preparation |
| PR-P | 2-Pyrrolidinecarboxylic acid | Propylene glycol | 1:1 | Failure of preparation |
| PR-B | 2-Pyrrolidinecarboxylic acid | 1,3-Butanediol | 1:1 | Failure of preparation |
| PR-X | 2-Pyrrolidinecarboxylic acid | Xylitol | 1:1 | Failure of preparation |
| PR-DS | 2-Pyrrolidinecarboxylic acid | D-Sorbitol | 1:1 | Failure of preparation |
| PR- MA2 | 2-Pyrrolidinecarboxylic acid | DL-Malic acid | 1:1 | Transparent viscous liquid |
| PR-PA | 2-Pyrrolidinecarboxylic acid | 1,5-Pentanedioic acid | 1:1 | Failure of preparation |
| PR-GA | 2-Pyrrolidinecarboxylic acid | Glycolic acid | 1:1 | Transparent liquid |
| PR-LA1 | 2-Pyrrolidinecarboxylic acid | Levulinic acid | 1:1 | Failure of preparation |
| PR- LA2 | 2-Pyrrolidinecarboxylic acid | Lactic acid | 1:1 | Transparent liquid |
| PR-CA | 2-Pyrrolidinecarboxylic acid | Citric acid | 1:1 | Transparent viscous liquid |
| B-LA2 | 1,3-Butanediol | Lactic acid | 1:1 | Transparent liquid |
| B-LA1 | 1,3-Butanediol | Levulinic acid | 1:1 | Transparent liquid |
| B-MA2 | 1,3-Butanediol | DL-Malic acid | 1:1 | Transparent liquid |
| B-CA | 1,3-Butanediol | Citric acid | 1:1 | Transparent liquid |
| B-GA | 1,3-Butanediol | Glycolic acid | 1:1 | Transparent liquid |
| B-PA | 1,3-Butanediol | 1,5-Pentanedioic acid | 2:1 | Transparent liquid |
| P-LA2 | Propylene glycol | Lactic acid | 1:1 | Transparent liquid |
| P-LA1 | Propylene glycol | Levulinic acid | 1:1 | Transparent liquid |
| P-MA2 | Propylene glycol | DL-Malic acid | 1:1 | Transparent liquid |
| P-CA | Propylene glycol | Citric acid | 1:1 | Transparent viscous liquid |
| P-GA | Propylene glycol | Glycolic acid | 1:1 | Transparent liquid |
| P-PA | Propylene glycol | 1,5-Pentanedioic acid | 2:1 | Transparent liquid |
| X-LA2 | Xylitol | Lactic acid | 1:1 | Transparent liquid |

| | | | | |
|---|---|---|---|---|
| X-LA1 | Xylitol | Levulinic acid | 1:1 | Transparent liquid |
| X-MA2 | Xylitol | DL-Malic acid | 1:1 | Transparent liquid |
| X-CA | Xylitol | Citric acid | 1:1 | Transparent liquid |
| X-GA | Xylitol | Glycolic acid | 1:1 | Transparent liquid |
| X-PA | Xylitol | 1,5-Pentanedioic acid | 2:1 | Transparent viscous liquid |

**Table S3.** List of the prepared ternary DESs based on X-GA.

| Number | The Third Component | Molar Ratio | Appearance at 45 °C |
|---|---|---|---|
| 1-1 | DL-Malic acid | 1:0:4 | Transparent liquid |
| 1-2 | DL-Malic acid | 1:1:3 | Transparent liquid |
| 1-3 | DL-Malic acid | 1:2:2 | Transparent liquid |
| 1-4 | DL-Malic acid | 1:3:1 | Transparent liquid |
| 1-5 | DL-Malic acid | 1:4:0 | Transparent liquid |
| 2-1 | Citric acid | 1:0:4 | Transparent liquid |
| 2-2 | Citric acid | 1:1:3 | Transparent liquid |
| 2-3 | Citric acid | 1:2:2 | Transparent liquid |
| 2-4 | Citric acid | 1:3:1 | Transparent liquid |
| 2-5 | Citric acid | 1:4:0 | Transparent liquid |
| 3-1 | Levulinic acid | 1:0:4 | Transparent liquid |
| 3-2 | Levulinic acid | 1:1:3 | Transparent liquid |
| 3-3 | Levulinic acid | 1:2:2 | Transparent liquid |
| 3-4 | Levulinic acid | 1:3:1 | Transparent liquid |
| 3-5 | Levulinic acid | 1:4:0 | Transparent liquid |
| 4-1 | Lactic acid | 1:0:4 | Transparent liquid |
| 4-2 | Lactic acid | 1:1:3 | Transparent liquid |
| 4-3 | Lactic acid | 1:2:2 | Transparent liquid |
| 4-4 | Lactic acid | 1:3:1 | Transparent liquid |
| 4-5 | Lactic acid | 1:4:0 | Transparent liquid |
| 5-1 | Propylene glycol | 1:0:4 | Transparent liquid |
| 5-2 | Propylene glycol | 1:1:3 | Transparent liquid |
| 5-3 | Propylene glycol | 1:2:2 | Transparent liquid |
| 5-4 | Propylene glycol | 1:3:1 | Transparent liquid |
| 5-5 | Propylene glycol | 1:4:0 | Transparent liquid |
| 6-1 | 1,5-Pentanedioic acid | 1:0:4 | Failure of preparation |
| 6-2 | 1,5-Pentanedioic acid | 1:1:3 | Failure of preparation |
| 6-3 | 1,5-Pentanedioic acid | 1:2:2 | Failure of preparation |
| 6-4 | 1,5-Pentanedioic acid | 1:3:1 | Transparent liquid |
| 6-5 | 1,5-Pentanedioic acid | 1:4:0 | Transparent liquid |
| 7-1 | Malonic acid | 1:0:4 | Failure of preparation |
| 7-2 | Malonic acid | 1:1:3 | Failure of preparation |
| 7-3 | Malonic acid | 1:2:2 | Transparent liquid |
| 7-4 | Malonic acid | 1:3:1 | Transparent liquid |
| 7-5 | Malonic acid | 1:4:0 | Transparent liquid |

**Table S4.** List of the prepared ternary DESs based on ChCl-MA2.

| Number | The Third Component | Molar Ratio | Appearance at 45 °C |
|---|---|---|---|
| 8-1 | Citric acid | 1:0:1 | Transparent liquid |
| 8-2 | Citric acid | 1:0.25:0.75 | Transparent liquid |
| 8-3 | Citric acid | 1:0.5:0.5 | Transparent liquid |
| 8-4 | Citric acid | 1:0.75:0.25 | Transparent liquid |
| 8-5 | Citric acid | 1:1:0 | Transparent liquid |
| 9-1 | Levulinic acid | 1:0:1 | Transparent liquid |
| 9-2 | Levulinic acid | 1:0.25:0.75 | Transparent liquid |
| 9-3 | Levulinic acid | 1:0.5:0.5 | Transparent liquid |
| 9-4 | Levulinic acid | 1:0.75:0.25 | Transparent liquid |
| 9-5 | Levulinic acid | 1:1:0 | Transparent liquid |
| 10-1 | Lactic acid | 1:0:1 | Transparent liquid |
| 10-2 | Lactic acid | 1:0.25:0.75 | Transparent liquid |
| 10-3 | Lactic acid | 1:0.5:0.5 | Transparent liquid |
| 10-4 | Lactic acid | 1:0.75:0.25 | Transparent liquid |
| 10-5 | Lactic acid | 1:1:0 | Transparent liquid |
| 11-1 | Propylene glycol | 1:0:1 | Failure of preparation |
| 11-2 | Propylene glycol | 1:0.25:0.75 | Transparent liquid |
| 11-3 | Propylene glycol | 1:0.5:0.5 | Transparent liquid |
| 11-4 | Propylene glycol | 1:0.75:0.25 | Transparent liquid |
| 11-5 | Propylene glycol | 1:1:0 | Transparent liquid |

| | | | |
|---|---|---|---|
| 12-1 | 1,5-Pentanedioic acid | 1:0:1 | Transparent liquid |
| 12-2 | 1,5-Pentanedioic acid | 1:0.25:0.75 | Transparent liquid |
| 12-3 | 1,5-Pentanedioic acid | 1:0.5:0.5 | Transparent liquid |
| 12-4 | 1,5-Pentanedioic acid | 1:0.75:0.25 | Transparent liquid |
| 12-5 | 1,5-Pentanedioic acid | 1:1:0 | Transparent liquid |
| 13-1 | Malonic acid | 1:0:1 | Transparent liquid |
| 13-2 | Malonic acid | 1:0.25:0.75 | Transparent liquid |
| 13-3 | Malonic acid | 1:0.5:0.5 | Transparent liquid |
| 13-4 | Malonic acid | 1:0.75:0.25 | Transparent liquid |
| 13-5 | Malonic acid | 1:1:0 | Transparent liquid |

**Table S5.** List of the prepared ternary DESs based on ChCl-CA.

| Number | The Third Component | Molar Ratio | Appearance at 45 °C |
|---|---|---|---|
| 14-1 | Levulinic acid | 1:0:1 | Transparent liquid |
| 14-2 | Levulinic acid | 1:0.25:0.75 | Transparent liquid |
| 14-3 | Levulinic acid | 1:0.5:0.5 | Transparent liquid |
| 14-4 | Levulinic acid | 1:0.75:0.25 | Transparent liquid |
| 14-5 | Levulinic acid | 1:1:0 | Transparent liquid |
| 15-1 | Lactic acid | 1:0:1 | Transparent liquid |
| 15-2 | Lactic acid | 1:0.25:0.75 | Transparent liquid |
| 15-3 | Lactic acid | 1:0.5:0.5 | Transparent liquid |
| 15-4 | Lactic acid | 1:0.75:0.25 | Transparent liquid |
| 15-5 | Lactic acid | 1:1:0 | Transparent liquid |
| 16-1 | Propylene glycol | 1:0:1 | Failure of preparation |
| 16-2 | Propylene glycol | 1:0.25:0.75 | Transparent liquid |
| 16-3 | Propylene glycol | 1:0.5:0.5 | Transparent liquid |
| 16-4 | Propylene glycol | 1:0.75:0.25 | Transparent liquid |
| 16-5 | Propylene glycol | 1:1:0 | Transparent liquid |
| 17-1 | 1,5-Pentanedioic acid | 1:0:1 | Transparent liquid |
| 17-2 | 1,5-Pentanedioic acid | 1:0.25:0.75 | Transparent liquid |
| 17-3 | 1,5-Pentanedioic acid | 1:0.5:0.5 | Transparent liquid |
| 17-4 | 1,5-Pentanedioic acid | 1:0.75:0.25 | Transparent liquid |
| 17-5 | 1,5-Pentanedioic acid | 1:1:0 | Transparent liquid |
| 18-1 | Malonic acid | 1:0:1 | Transparent liquid |
| 18-2 | Malonic acid | 1:0.25:0.75 | Transparent liquid |
| 18-3 | Malonic acid | 1:0.5:0.5 | Transparent liquid |
| 18-4 | Malonic acid | 1:0.75:0.25 | Transparent liquid |
| 18-5 | Malonic acid | 1:1:0 | Transparent liquid |

**Table S6.** List of the prepared ternary DESs based on B-MA2.

| Number | The Third Component | Molar Ratio | Appearance at 45 °C |
|---|---|---|---|
| 19-1 | Citric acid | 1:0:1 | Transparent liquid |
| 19-2 | Citric acid | 1:0.25:0.75 | Transparent liquid |
| 19-3 | Citric acid | 1:0.5:0.5 | Transparent liquid |
| 19-4 | Citric acid | 1:0.75:0.25 | Transparent liquid |
| 19-5 | Citric acid | 1:1:0 | Transparent liquid |
| 20-1 | Levulinic acid | 1:0:1 | Transparent liquid |
| 20-2 | Levulinic acid | 1:0.25:0.75 | Transparent liquid |
| 20-3 | Levulinic acid | 1:0.5:0.5 | Transparent liquid |
| 20-4 | Levulinic acid | 1:0.75:0.25 | Transparent liquid |
| 20-5 | Levulinic acid | 1:1:0 | Transparent liquid |
| 21-1 | Lactic acid | 1:0:1 | Transparent liquid |
| 21-2 | Lactic acid | 1:0.25:0.75 | Transparent liquid |
| 21-3 | Lactic acid | 1:0.5:0.5 | Transparent liquid |
| 21-4 | Lactic acid | 1:0.75:0.25 | Transparent liquid |
| 21-5 | Lactic acid | 1:1:0 | Transparent liquid |
| 22-1 | Propylene glycol | 1:0:1 | Transparent liquid |
| 22-2 | Propylene glycol | 1:0.25:0.75 | Transparent liquid |
| 22-3 | Propylene glycol | 1:0.5:0.5 | Transparent liquid |
| 22-4 | Propylene glycol | 1:0.75:0.25 | Transparent liquid |
| 22-5 | Propylene glycol | 1:1:0 | Transparent liquid |
| 23-1 | 1,5-Pentanedioic acid | 1:0:1 | Failure of preparation |
| 23-2 | 1,5-Pentanedioic acid | 1:0.25:0.75 | Transparent liquid |
| 23-3 | 1,5-Pentanedioic acid | 1:0.5:0.5 | Transparent liquid |
| 23-4 | 1,5-Pentanedioic acid | 1:0.75:0.25 | Transparent liquid |
| 23-5 | 1,5-Pentanedioic acid | 1:1:0 | Transparent liquid |

| | | | |
|---|---|---|---|
| 24-1 | Malonic acid | 1:0:1 | Transparent liquid |
| 24-2 | Malonic acid | 1:0.25:0.75 | Transparent liquid |
| 24-3 | Malonic acid | 1:0.5:0.5 | Transparent liquid |
| 24-4 | Malonic acid | 1:0.75:0.25 | Transparent liquid |
| 24-5 | Malonic acid | 1:1:0 | Transparent liquid |
